# Editorial: Dietary acrylamide in human health

**DOI:** 10.3389/fnut.2024.1446690

**Published:** 2024-06-25

**Authors:** Federica Laguzzi, Tommaso Filippini, Ana Virgolino

**Affiliations:** ^1^Unit of Cardiovascular and Nutritional Epidemiology, Institute of Environmental Medicine, Karolinska Institutet, Stockholm, Sweden; ^2^Environmental, Genetic and Nutritional Epidemiology Research Center (CREAGEN) - Section of Public Health, Department of Biomedical, Metabolic and Neural Sciences, University of Modena and Reggio Emilia, Modena, Italy; ^3^EnviHeB Lab, Institute of Environmental Health, Faculty of Medicine, University of Lisbon, Lisbon, Portugal

**Keywords:** acrylamide, contaminant, carcinogen, genotoxic, human health, dietary exposure

Acrylamide, a food processing contaminant, has raised concerns due to potential health risks to humans. It is known to be formed mainly in starch-rich foods, cooked or processed at high temperatures (above 120°C) in the absence of moisture, via the Maillard reaction when a reduced carbohydrate (glucose and fructose) combines with the amino acid asparagine (1). Moreover, evidence suggests that acrylamide may also form from the degradation of proteins and fats.

Acrylamide is present in a large variety of commonly consumed products such as coffee, potato-derived products, and cereal-derived products, including breakfast cereals, bread, and biscuits. Except for boiling, acrylamide may form with the most common cooking methods e.g., grilling, baking, roasting, and frying. Additionally, several chemical and physical factors, namely pH, can affect acrylamide formation during food cooking or processing. By improving our understanding of how to modify these factors, it is possible to significantly reduce acrylamide formation in cooked and processed foods, thereby mitigating potential health risks associated with acrylamide.

All ages and groups are exposed to dietary acrylamide with increasing trend of the recent years (2). Animal and mechanistic studies suggested that acrylamide is a probable carcinogen to humans (3), although epidemiological evidence is still limited and controversial (3–5). Occupational studies further support that acrylamide is a potential neurotoxicant. Neurotoxicity is considered the most sensitive non-neoplastic endpoint particularly for toddlers and children due to a small margin of exposure in these age groups (6, 7). Furthermore, emerging evidence shows that acrylamide might also be associated with other chronic diseases in humans (8, 9).

Efficient cell reparative or detoxication mechanisms in humans could also have a protective role from acrylamide exposure. In animal experiments, the toxicity is induced due to saturation of the detoxification mechanisms as well as not all the damages are repaired since the reparative capacity is exceeded. As suggested in some previous research, vitamins and bioactive substances in food might also play a protective role in humans (10).

This Research Topic aimed to improve knowledge on dietary acrylamide and its health effects focusing on studies investigating the levels of dietary acrylamide in certain groups, analyzing the mechanisms underlying toxicity in animals and humans, and investigating methods to reduce exposure.

Navruz-Varli and Mortaş investigated cooking methods and pre-treatments on acrylamide content in fried potatoes comparing traditional deep frying with oven and air frying. Before cooking, potatoes were pre-treated using running water for 30 s (washing) or within room temperature water for 10 min (soaking). After washing, the highest levels of acrylamide content were found in the air fryer followed by deep and oven frying. Soaking demonstrated lower acrylamide formation in all cooking methods, especially in deep frying, showing the lowest acrylamide content compared to both air and oven frying. Despite the spread of air frying as an alternative cooking method in recent years, the study highlights that acrylamide formation cannot be greatly decreased, independently from pre-treatment, while oven frying showed on average lower acrylamide content. Finally, soaking showed to be a primary pre-treatment to drop down acrylamide formation in traditional deep frying.

Using animal models, Chen et al. studied how kiwifruit polysaccharide, which has been shown to have a favorable effect on biological and metabolic functions, can have a protective effect against acrylamide induced disorders. To do so, the authors assessed changes in gut microbiota and serum metabolites in acrylamide exposed mice. The results show that the kiwifruit polysaccharide can have protective benefits against acrylamide-induced toxicity by improving body features and having a hepatoprotective effect, potentially through the gut-liver axis. The restauration of the gut microbiota was associated with an increase in microbial diversity with a proliferation of beneficial bacteria.

Homayoonfal et al. revised the available evidence regarding the change in microRNA (miRNA) regulation profiles after acrylamide intake and the mechanisms behind its toxicity. The focus was to assess the mediating effects of miRNAs that regulate various cellular and molecular processes, on acrylamide's hazardous potential. The results suggest that acrylamide can change and dysregulate miRNA profiles in different tissues, as shown in both animal and cell line models, potentially having carcinogenic effects and influencing disease progression by affecting signaling pathways. A specific miRNA profile associated with exposure to acrylamide intake might constitute a valuable biomarker for assessing exposure, diagnosis, and potential therapeutic approaches. Even so, the authors highlight the importance of determining not only a miRNA profile associated with acrylamide-induced cancer and degenerative diseases but also the relevance of such profile in non-cancerous disorders.

In the study of Vryonidis et al. acrylamide intake and hemoglobin (Hb) adducts, validated biomarkers of acrylamide in blood, were assessed in Swedish adolescents aged 10–21 years. Acrylamide intake was estimated using a 24 h dietary recall method combining consumption of food groups with higher acrylamide content. Both acrylamide and glycidamide Hb-adducts were measured and further used to estimate acrylamide intake to be compared with dietary recall. Authors found similar intake of acrylamide among study participants, showing that foods most contributing to acrylamide intake in these Swedish adolescents are fried potatoes, followed by bakery, cakes and bread, with limited to null contribution of coffee due different dietary habits compared to adults. The study confirmed that also the measured levels of acrylamide Hb-adducts were similar among participants except for the higher levels in daily smokers compared to occasionally, former and never smokers. Acrylamide intake estimated using dietary recall was generally double compared to Hb-adducts, with low but still positive correlation. Finally, the study estimated cancer risk using the margin of exposure approach and highlighted the potential health concerns in the study participants, indicating the need to further monitor and lower acrylamide exposure, particularly in non-adult population.

In conclusion, the results obtained by the studies published on this Research Topic on acrylamide are forming important new scientific basis to understand better its biological and molecular mechanisms, identify susceptible groups, and implement exposure-limiting methods. However, further research is urgently needed to contribute to risk assessment, safety measures, exposure monitoring, and a deeper understanding of its potential health effects.

## Author contributions

FL: Conceptualization, Writing – original draft, Writing – review & editing. TF: Conceptualization, Writing – original draft, Writing – review & editing. AV: Conceptualization, Writing – original draft, Writing – review & editing.

## References

[1] Tareke E Rydberg P Karlsson P Eriksson S Tornqvist M. Analysis of acrylamide, a carcinogen formed in heated foodstuffs. J Agric Food Chem. (2002) 50:4998–5006. 10.1021/jf020302f 12166997

[2] Poteser M Laguzzi F Schettgen T Vogel N Weber T Zimmermann P. Time trends of acrylamide exposure in Europe: combined analysis of published reports and current HBM4EU studies. Toxics. (2022) 10:481. 10.3390/toxics10080481 36006160 PMC9415789

[3] World Health Organization International International Agency for Research on Cancer. IARC monographs on the evaluation of carcinogenic risks to humans. IARC working group on the evaluation of carcinogenic risks to humans: some industrial chemicals. Lyon, 15-22 February 1994. IARC Monogr Eval Carcinog Risks Hum. (1994) 60:1–560.7869568

[4] Adani G Filippini T Wise LA Halldorsson TI Blaha L Vinceti M. Dietary intake of acrylamide and risk of breast, endometrial, and ovarian cancers: a systematic review and dose-response meta-analysis. Cancer Epidemiol Biomarkers Prev. (2020) 29:1095–106. 10.1158/1055-9965.EPI-19-1628 32169997

[5] Filippini T Halldorsson TI Capitão C Martins R Giannakou K Hogervorst J. Dietary acrylamide exposure and risk of site-specific cancer: a systematic review and dose-response meta-analysis of epidemiological studies. Front Nutr. (2022) 9:875607. 10.3389/fnut.2022.875607 35548558 PMC9082595

[6] Lindeman B Johansson Y Andreassen M Husøy T Dirven H Hofer T. Does the food processing contaminant acrylamide cause developmental neurotoxicity? A review and identification of knowledge gaps. Reprod Toxicol. (2021) 101:93–114. 10.1016/j.reprotox.2021.02.006 33617935

[7] El-Zakhem Naous G Merhi A Daher R Mroueh M Abboud MI Taleb RI. Carcinogenic and neurotoxic risks of acrylamide consumed through bread, kaak, toast, and crackers among the Lebanese Population. Regul Toxicol Pharmacol. (2022) 132:105192. 10.1016/j.yrtph.2022.105192 35654311

[8] Buyukdere Y Akyol A. From a toxin to an obesogen: a review of potential obesogenic roles of acrylamide with a mechanistic approach. Nutr Rev. (2023) 82:128–42. 10.1093/nutrit/nuad041 37155834 PMC10711450

[9] Hogervorst J Virgolino A Halldorsson TI Vinceti M Åkesson A Leander K. Maternal acrylamide exposure during pregnancy and fetal growth: A systematic review and dose-response meta-analysis of epidemiological studies. Environ Res. (2022) 213:113705. 10.1016/j.envres.2022.113705 35724727

[10] Govindaraju I Sana M Chakraborty I Rahman MH Biswas R Mazumder N. Dietary acrylamide: a detailed review on formation, detection, mitigation, and its health impacts. Foods. (2024) 13:556. 10.3390/foods13040556 38397533 PMC10887767

